# Agriculture and crop dispersal in the western periphery of the Old World: the Amazigh/Berber settling of the Canary Islands (ca. 2nd–15th centuries ce)

**DOI:** 10.1007/s00334-023-00920-6

**Published:** 2023-06-22

**Authors:** Jacob Morales, Claudia Speciale, Amelia Rodríguez-Rodríguez, Pedro Henríquez-Valido, Efrain Marrero-Salas, Juan Carlos Hernández-Marrero, Rosa López, Teresa Delgado-Darias, Jenny Hagenblad, Rosa Fregel, Jonathan Santana

**Affiliations:** 1 TARHA Research Group, Department of Historical Sciences, University of Las Palmas de Gran Canaria, C. de Pérez del Toro 1, 35004 Las Palmas de Gran Canaria, Las Palmas Spain; 2 IPHES-CERCA, Catalan Institute of Human Paleoecology and Social Evolution, Zona Educacional 4 - Campus Sescelades URV (Edifici W3), 43007 Tarragona, Spain; 3 Department of Geography and History, University of La Laguna, Campus de Guajara, Las Chumberas, La Laguna, Tenerife Spain; 4 Archaeological Museum of La Gomera, C. Torres Padilla 6, 38800 San Sebastián de La Gomera, Santa Cruz de Tenerife Spain; 5 Arenisca Arqueología y Patrimonio, c. Salvia 3, La Oliva, CP 35660 Fuerteventura, Spain; 6 Archaeological Museum El Museo Canario, Calle del Dr. Verneau 2, 35001 Las Palmas de Gran Canaria, Las Palmas Spain; 7 Department of Physics, Chemistry and Biology, Linköping University, Olaus Magnus väg, Linköping, 583 30 Sweden; 8 Evolution, Population Genetics and Paleogenomics Research Group, Department of Biochemistry, Microbiology, Cell Biology and Genetics, University of La Laguna, Pabellón de Gobierno, C/ Molinos de Agua s/n, 38200 La Laguna, Santa Cruz de Tenerife Spain

**Keywords:** Canary Islands, Pre-hispanic period, Amazigh, Archaeobotany, Agriculture, Human colonisation, Wild plant gathering

## Abstract

The Canary Islands were settled ca. 1,800 years ago by Amazigh/Berber farming populations originating in North Africa. This historical event represents the last and westernmost expansion of the Mediterranean farming package in Antiquity, and investigating it yields information about crop dispersal along the periphery of the Mediterranean world around the turn of the first millennium ce. The current study focuses on archaeobotanical evidence recorded in a series of pre-Hispanic/Amazigh sites of the Canary Islands (ca. 2nd–15th centuries ce). It offers new, unpublished archaeobotanical findings and direct radiocarbon datings of plant remains from the different islands. The general goal is to gain a better grasp of how the first settlers of the Canary Islands adapted their farming activities to the different natural conditions of each island. The results suggest a shared crop ‘package’ throughout the islands since at least the 3rd–5th centuries ce. This set of plants was likely introduced from north-western Africa and consists of *Hordeum vulgare* (hulled barley), *Triticum durum* (durum wheat), *Lens culinaris* (lentil), *Vicia faba* (broad bean), *Pisum sativum* (pea), and *Ficus carica* (fig). The crop ‘package’ probably arrived in a single episode during the initial colonisation and was not followed by any other plants. Subsequent to the initial settling and until the arrival of the European seafarers, the islands remained isolated from each other and from the outside world, a condition that over time led to a decline in crop diversity in all of the islands except Gran Canaria.

**Supplementary Information:**

The online version contains supplementary material available at 10.1007/s00334-023-00920-6.

## Introduction

The Canary Islands form an archipelago of volcanic origin off the north-western African coast with its main islands covering a surface area of less than 7,500 km^2^. The archipelago is environmentally diverse marked by a rich native flora and fauna (Afonso 1997; Arechavaleta Hernandéz et al. 2010). The islands were permanently settled only about 1,800 years ago, 2nd to 5th centuries (c.) ce by communities sharing genetic, linguistic, and other cultural traits with the current Amazigh/Berber populations of the Maghreb, suggesting that the early settlers originated in north-western Africa (Springer Bunk 2015–2016; Rodríguez-Varela et al. 2017; Fregel et al. 2019; Blench 2021). Popularly known as *Guanches* (a name referring only to the early inhabitants of Tenerife), the settlers populated the archipelago with little or no inter-island or mainland contact until the arrival of European seafarers in the 14th–15th c. ce. Although there is evidence of a seasonal camp occupied by Roman fishers and gatherers of *Stramonita haemastoma* (red-mouthed rock shell) for Tyrian purple on the Islet of Lobos in the 1st c. bce, the current data indicate that these early groups did not occupy any of the islands permanently (Del Arco Aguilar et al. 2017).

The subsistence of the settlers derived from a combination of gathering wild plants and molluscs with fishing. However, the successful colonisation of the archipelago was only rendered possible by the introduction of crops and domesticated animals from the mainland. This largely neglected and understudied spread of crops associated with the Amazigh populations of the Maghreb during the 1st millennium ce represents the last and westernmost expansion of the Mediterranean farming package in Antiquity.

The settlers brought a limited set of Mediterranean crops: *Hordeum vulgare* ssp. *vulgare* (six-row hulled barley), *Triticum durum* (durum wheat), *Lens culinaris* (lentil), *Vicia faba* (broad bean), *Pisum sativum* (pea) and *Ficus carica* (fig) (Morales et al. 2017). There is also evidence they introduced *Capra hircus* (goat), *Ovis aries* (sheep), *Sus domestica* (pig), *Canis familiaris* (dog) and possibly *Felis catus* (cat) (Pais Pais 1996; Castellano Alonso et al. 2016; Hernández-Marrero et al. 2016) alongside the accidental arrival of the house mouse, insect pests and weeds (Morales et al. 2009; Rando et al. 2012; Henríquez-Valido et al. 2020).

This paper aims to offer a first framework for understanding the origin and spread of agriculture in the Canary Islands by critically reviewing the available archaeobotanical evidence of the pre-Hispanic period (ca. 2nd to 15th c. ce). In addition, this study offers new unpublished archaeobotanical data and direct radiocarbon datings of plant remains from each of the islands. Seed and fruit remains recovered during excavations of archaeological sites provide primary and direct information about crops and agricultural activities as these types of plant macroremains can be directly dated by radiocarbon methods and their identification is much simpler than other proxies such as pollen or phytoliths. Hence, the main objectives of the current study are (1) to characterise the domestic and wild plant resources serving the pre-Hispanic populations for subsistence, and (2) to examine the spatial-temporal variations in cultivation activities among the different islands of the archipelago.

## Environmental setting and cultural context

**Fig. 1:**
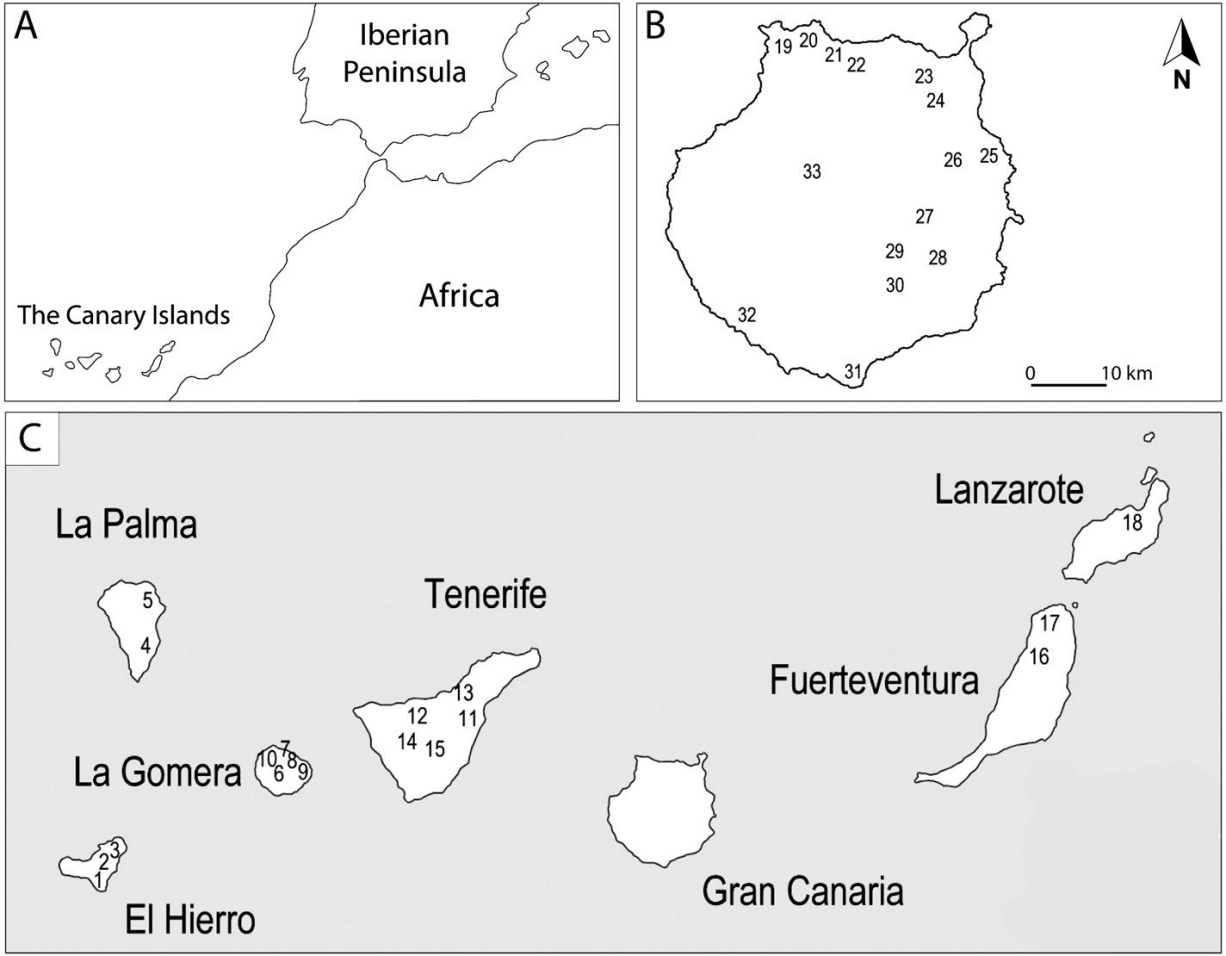
Map of the Canary Islands indicating, **A** the position of the archipelago in relation to Europe and Africa, **B** the archaeological sites in Gran Canaria cited in the text, and **C** archaeological sites in the archipelago cited in the text: (1) La Lajura; (2) Hoya del Zarzal; (3) Afotasa; (4) Belmaco; (5) El Tendal; (6) Alto del Garajonay; (7) Cañada de la Gurona; (8) Cueva Honda; (9) Lomito del Medio; (10) Sobrado de los Gomeros; (11) Chinguaro; (12) Don Gaspar; (13) Bencomo; (14) Chasogo; (15) Cruz de Tea; (16) Cueva de Villaverde; (17) Punta Mallorquín; (18) Fiquinineo; (19) Playa Chica; (20) Cueva Pintada; (21) Cenobio de Valerón; (22) La Montañeta; (23) La Cerera; (24) El Tejar; (25) Lomo los Melones; (26) Cendro; (27) Cuevas Muchas-Guayadeque; (28) Ermita de San Antón; (29) Temisas; (30) La Fortaleza; (31) Dunas de Maspalomas; (32) Lomo los Gatos; (33) El Álamo-Acusa

The Canarian archipelago consists of seven major islands and several islets (Fig. 1). Their surface areas range from ca. 2,034 km^2^ (Tenerife) to 268 km^2^ (El Hierro). The two known as the eastern islands (Fuerteventura and Lanzarote) lack high mountains and are very arid. The western islands (Gran Canaria, Tenerife, La Gomera, La Palma and El Hierro), in turn, range in maximum altitude from 1,501 m (El Hierro) to 3,718 m (Tenerife), conditions that generate a higher rate of precipitation and greater ecological diversity (Afonso 1997).

The chronology of the pre-Hispanic period has recently been revised in light of new radiocarbon datings and critical analyses of prior data (Velasco-Vázquez et al. 2020; Alberto-Barroso et al. 2022a, 2022b; Pardo-Gordó et al. 2022). The earliest reliable absolute dates come from Lanzarote, the easternmost island, where the dating range of an ovicaprid bone extends from the 1st c. bce to the 4th c. ce (Atoche Peña and Ramírez Rodríguez 2017). Datings from the westernmost islands point to a human presence there from at least the 3rd–5th c. ce (Morales et al. 2017; Sánchez-Cañadillas et al. 2021) suggesting that all the islands were rapidly colonised subsequent to those of the east, closest to the African mainland. Although no additional colonisation waves have been recorded, certain researchers interpret new arrivals based on changes in material culture and funerary practices (Martín de Guzmán 1984; Velasco-Vázquez et al. 2021).

The technology of the pre-Hispanic indigenous population was characterised by the lack of metal tools and a rich lithic industry comprising volcanic rocks such as basalt and obsidian (Rodríguez-Rodríguez et al. 2017; Rodríguez-Rodríguez and Francisco-Ortega 2019). Tools and other materials were likewise fashioned from animal bones, leather, seashells and plants (Rodríguez-Rodríguez and Navarro-Mederos 1999; Vidal-Matutano et al. 2021). Pottery containers were common, bearing decorative patterns characteristic of both archipelago and even single island groups (Del Pino Curbelo and Rodríguez-Rodríguez 2017). The archaeological sites of the western islands consist of a high proportion of caves and rock shelters and a lesser number of seasonal open-air shelters. Open-air and cave settlements are the most common type in Gran Canaria and the eastern islands (Navarro-Mederos 1997). There is little evidence of social stratification throughout most of the archipelago, and it is likely that the number of inhabitants on each island was continuously low (Mederos Martín 2019). The situation of Gran Canaria, however, appears to differ as it reveals large settlements, cemeteries, and complex cave-granaries suggesting a large population with more complex social organisation (Onrubia Pintado 2003; Velasco-Vázquez et al. 2021).

Palaeoecological records such as fossil pollen and wood charcoals from the archaeological sites signal that the natural vegetation of the islands was modified after the arrival of the first settlers (de Nascimento et al. 2020). Pollen research from the western islands suggests forest clearing and changes in the types of species, at times resulting in a greater frequency of wildfires (Nogué et al. 2013; de Nascimento et al. 2016; Fernández-Palacios et al. 2016). Wood charcoal analyses in certain cases also reveal significant changes in the vegetation (Machado Yanes 1996; Machado Yanes et al. 1997).

## Materials and methods

The current study collected archaeobotanical data from a total of 28 sites and reviewed all the relevant literature. Apart from Cueva de Don Gaspar (Tenerife), a site analysed by Maria Hopf in the 1980s, all the studies were carried out by participants (JM, PHV and CS) in this paper. Not all sites benefitted from the same sampling and processing methods. As sample sizes are highly variable, the site to site comparisons are mainly based on the presence/absence of crops rather than total numbers.

Also included here is unpublished evidence from five new sites, notably the first archaeobotanical analyses from the islands of Lanzarote and Fuerteventura. Most samples (Tables 1 and 2) were processed with a siraf-type water flotation system before collecting the plant materials with 0.25 mm sieves. Seeds and other plant macroremains were separated and identified with a stereomicroscope (8×− 80×). Charring mostly accounted for the preservation of the plant fossils. Dry sieving served to collect most of the desiccated items from the cave-granaries of Gran Canaria. A reference collection of modern seeds at the Laboratory of Archaeology (University of Las Palmas de Gran Canaria) and seed atlases (Cappers et al. 2009) served to identify the species. Certain plant remains were determined and recorded based on their anatomical and taxonomical characteristics. Each value corresponds to a complete item (seed, fruit, rachis segment, and so on) or fragments bearing a key feature such as an embryo. The nomenclature of the crop plants follows the traditional classification (Zohary et al. 2012), whereas the terms advanced by Arechavaleta Hernandéz et al. (2010) and the updated data of the catalogue of the WFO (2022+) served specifically for Canarian wild plants.

**Table 1:**
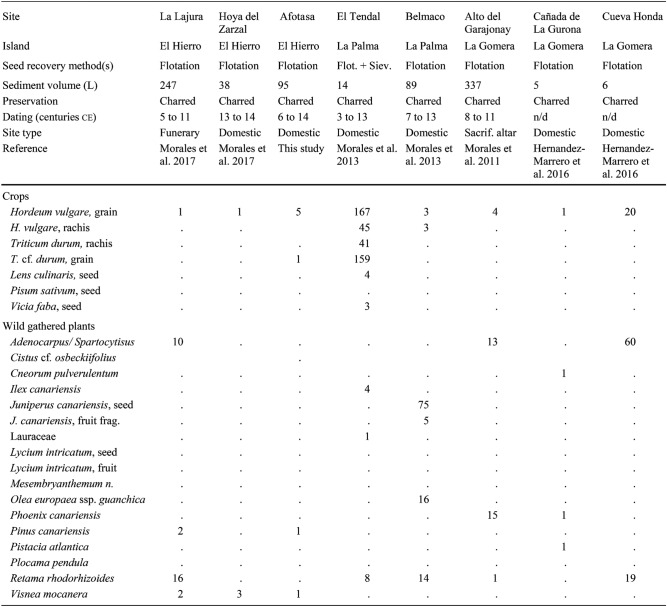

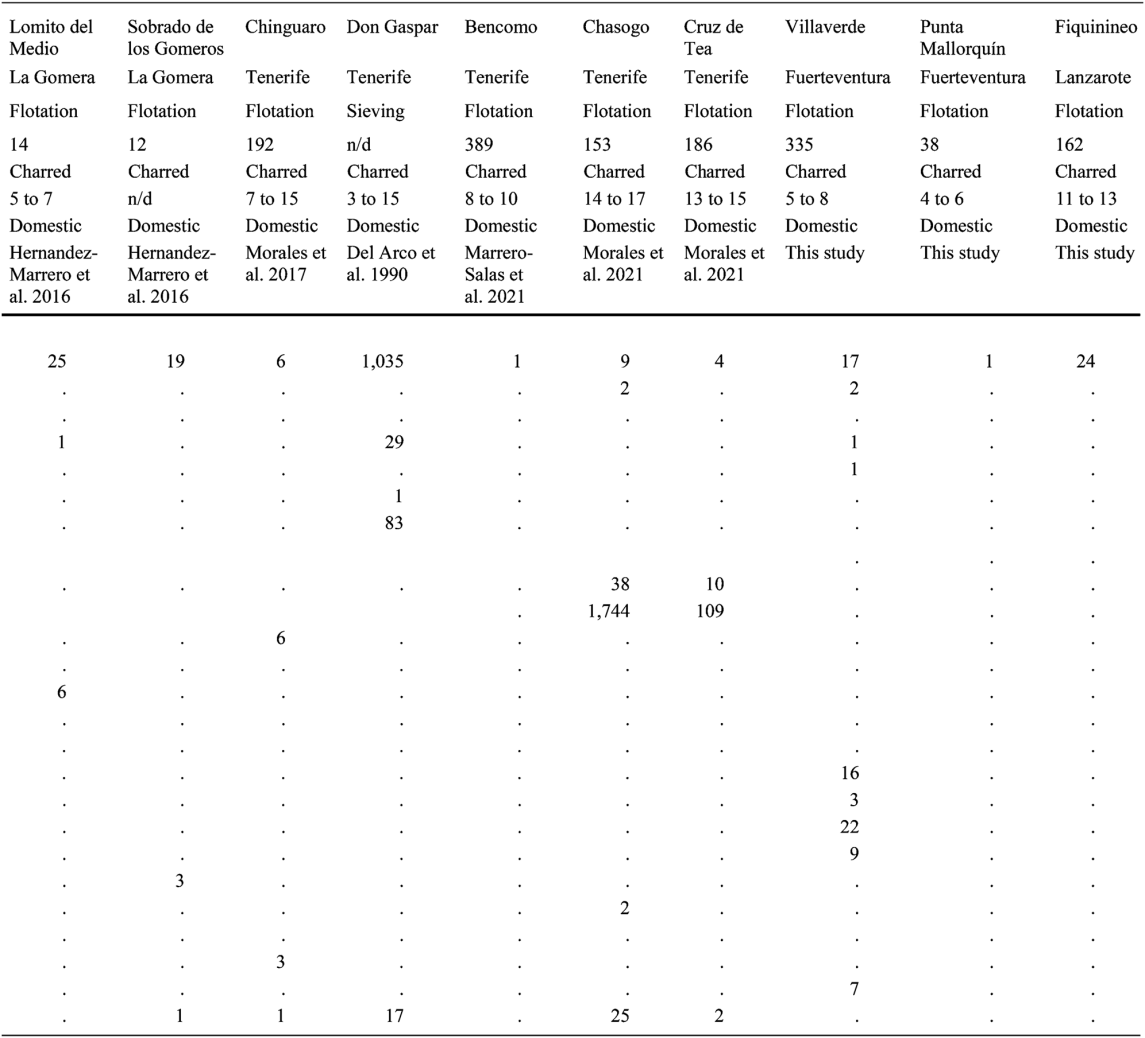
List of the values of plant macroremains collected at the sites mentioned in the text from the islands of El Hierro, La Palma, La Gomera, Tenerife, Fuerteventura, Lanzarote (excluding Gran Canaria)

**Table 2:**
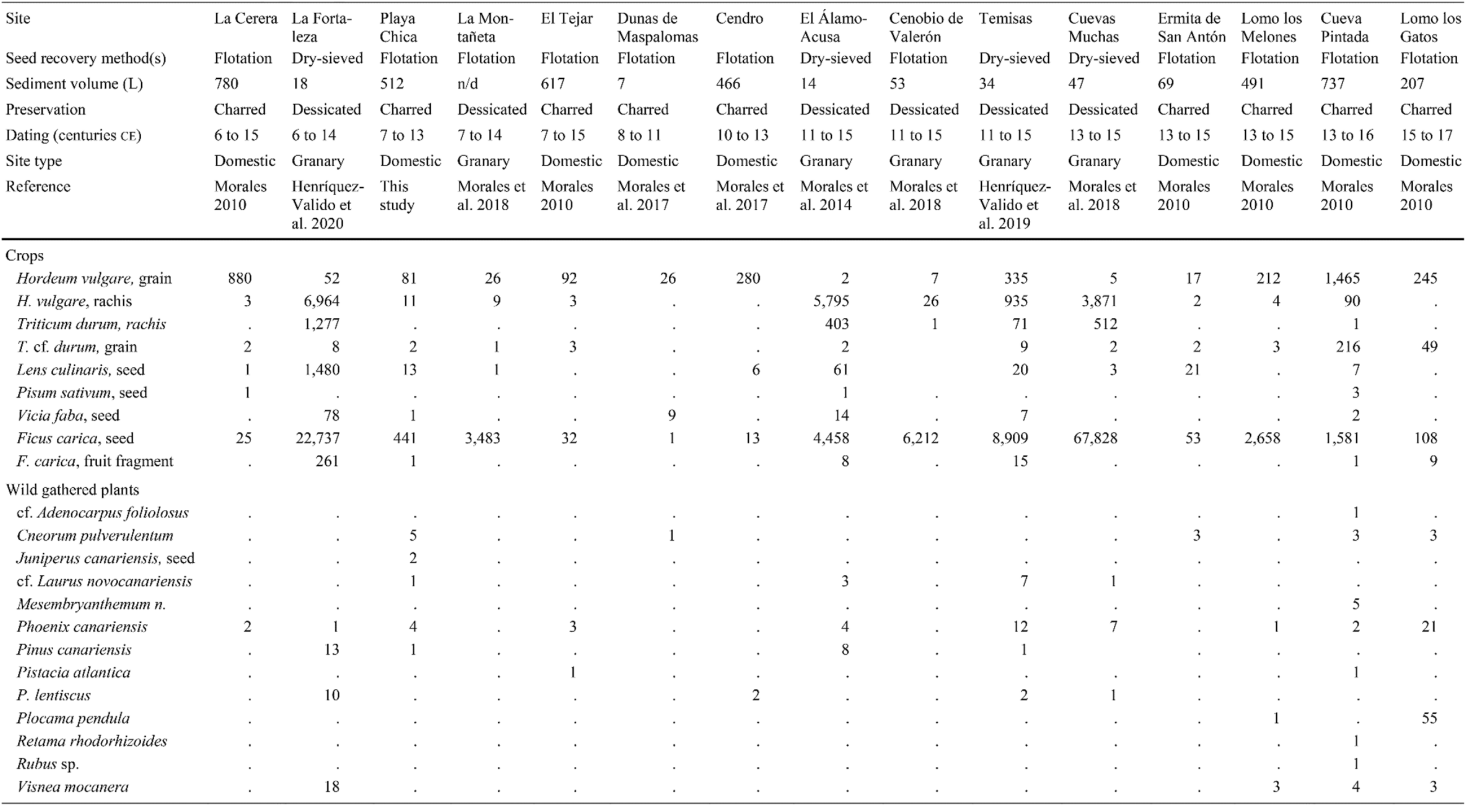
List of the values of plant macroremains collected at the sites on Gran Canaria

The results are organised by island, from east to west. Data concerning weeds and ruderals are excluded as the focus here is exclusively on crops and wild native plants bearing historical and ethnographic evidence of consumption (Morales and Gil 2014a). Table 1 lists the individual results for the sites in each island except for Gran Canaria, which is in Table 2. Table 3, in turn, summarises the results of each island. All available information, including radiocarbon datings, sampling methods, sediment volumes and plant remain states of preservation are listed in the tables. Certain sites with evidence of crops, albeit not benefiting from direct radiocarbon datings, are nevertheless included as finds of other archaeological materials clearly manifest Amazigh/Berber occupations.

**Table 3:**
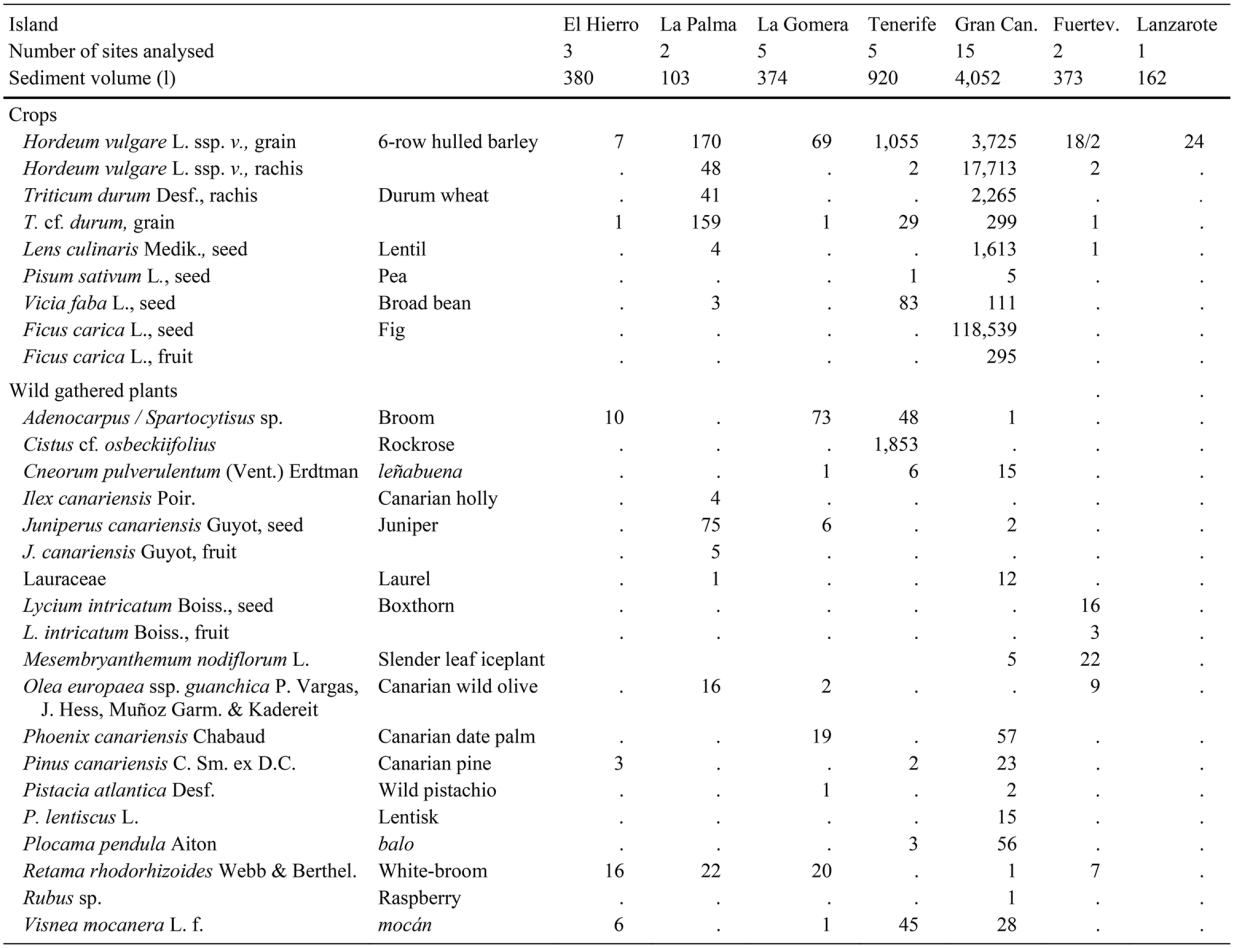
List of plant macroremains collected on each island of the archipelago

This study also resorted to old texts penned in the 14th–15th c. ce by European explorers referring to indigenous plant use. Although biased and at times contradictory, this information offers compelling details as to crop diversity and plant use during the period of contact with the Europeans (Morales and Gil 2014a).

Table 4 compiles the AMS datings of crop seeds from the different islands. It includes 10 unpublished dates carried out on domesticated plant remains from sites throughout the seven islands. This represents the first assemblage of evidence of pre-Hispanic agriculture on all the islands of the archipelago. The table also includes new radiocarbon analyses of remains of four humans from Gran Canaria with *F. carica* seeds trapped in their dental caries, thus offering a direct timeframe of the cultivation and consumption of this fruit.

## Results

### Lanzarote

The sole archaeobotanical evidence of agriculture in Lanzarote comes from the site of Fiquinineo-La Peña de las Cucharas (de León Hernández et al. 2016; Marrero-Salas et al. 2017) as the archaeological excavations on Lanzarote prior to Fiquinineo did not sample sediments for plant remains. Charred *Hordeum* grains from Fiquinineo yielded a radiocarbon dating ranging from the 11th to the 13th c. ce. No other plant remains were recovered among the samples. Moreover, written sources by the first Europeans arriving in Lanzarote in the early 15th c. ce state that this island’s indigenous population (known as *Majos*) only cultivated *Hordeum* (Bontier and Le Verrier 1980).

### Fuerteventura

Evidence of crops from Fuerteventura stem from two recent excavations: Cueva de Villaverde and Punta Mallorquín. The first yielded charred *Hordeum*, *Triticum* and *Lens* as well as seeds of edible wild plants such as *Olea europaea* ssp. *guanchica* (Canarian wild olive), *Lycium intricatum* (boxthorn) and *Mesembryanthemum nodiflorum* (slender leaf iceplant). The two ^14^ C datings for Villaverde were carried out on *Triticum* (5th–6th c. ce) and *Hordeum* grains (7th–8th c. ce). The dating of Punta Mallorquín (4th–6th c. ce), in turn, was carried out on the bone of a domestic animal. Written records from the early 15th c. ce note that Fuerteventura’s indigenous population (also known as *Majos*) did not cultivate any crops and only consumed domestic animal meat and other products, notably fish and molluscs (Bontier and Le Verrier 1980).

### Gran Canaria

Gran Canaria has benefitted throughout the last two decades from extensive archaeobotanical research as well as systematic sediment sampling and flotation (Morales 2010; Morales et al. 2017) and plant macroremains analyses have been carried out at 15 settlements and cave-granaries. The latter are artificial caves set in inaccessible or naturally fortified locations such as cliffs serving for storage (Morales et al. 2014; Henríquez-Valido et al. 2019, 2020). The conditions of preservation of finds in these caves are exceptional, notably the desiccation of plant remains.

The crops from the archaeological sites of Gran Canaria include *Hordeum*, *T. durum*, *L. culinaris*, *V. faba*, *P. sativum* and *F. carica* and all date from the earliest occupations (Morales et al. 2017). Direct ^14^ C datings of all these species (except *P. sativum*) further corroborate their presence since the 6th–7th c. ce. Furthermore, a series of datings of human individuals with desiccated *Ficus* seed remains embedded in their dental caries yield a timeframe ranging from the 7th to the 13th c. ce (Morales and Delgado-Darias 2007).

The written sources from the period of contact with the Europeans point to the cultivation of *Hordeum*, *Triticum*, *V. faba* and *F. carica* by the indigenous population (known as *Canarios*). *Hordeum* was referred to as *azomatan* whereas *F. carica* fruits were known as *arehormaze* when unripe and *tehaunenen* when fully ripe and dried (de Abreu Galindo 1977; Morales and Gil 2014b). Several of these early authors indicate that a large portion of the cereal production was carried out in artificially irrigated fields along valley bottoms (de Abreu Galindo 1977).

Evidence of wild plants in Gran Canaria is rare at archaeological sites as they probably did not play a significant role in the diet (Henríquez-Valido et al. 2020). Exceptions are fruits of *Phoenix canariensis* (Canarian date palm) and *Visnea mocanera* (*mocán*). The historical written narratives also cite the consumption of these plants at the time of contact with European explorers (Morales and Gil 2014a).

### Tenerife

There is scarce direct archaeobotanical evidence of the agricultural practices of Tenerife’s indigenous population. The only information of this type stems from four sites (Chinguaro, Bencomo, Chasogo and Cruz de Tea) where sediments were systematically sampled and processed by flotation. In these cases the only evidence of crops is that of a few *Hordeum* grains (Morales et al. 2017, 2021; Marrero-Salas et al. 2021), the ^14^ C datings of which reveal ranges from the 7th–9th to 13th–17th c. ce (Table 4).

The corpus in Tenerife also includes finds from dry-sieved (ca. 2 mm mesh) soil samples from the cave-site of Cueva de Don Gaspar, which yielded many seeds of *Hordeum*, *Triticum*, *V. faba*, and possibly *P. sativum.* The *Hordeum*, *Triticum* and pulses were collected from the lower layers, whereas the upper layers only contained *Hordeum* and wild plants, a tendency suggesting a decrease in crop diversity (Del Arco Aguilar et al. 1990). No direct ^14^ C dates on seeds are available for this site and there are only two standard ^14^ C dates, both from the 1st millennium ce, on non-identified wood charcoals.

European explorers in the 15th c. ce cite *Hordeum*, *Triticum*, *V. faba* and *P. sativum* cultivation by Tenerife’s indigenous population (known as *Guanches*). They recorded the names given to the crops: *Hordeum* (the most important) was *tamo*, *Triticum* was *yrichen* and *V. faba*/*P. sativum* was *hacichey* (de Abreu Galindo 1977; de Espinosa 1980).

Wild plants were a key resource, at least between the 13th–17th c. ce, as indicated by finds from the sites of Chasogo and Cruz de Tea. The most common edible taxa are seeds of *Cistus* sp. (rockrose*), mocán*, and *Pinus canariensis* (pine) (Morales et al. 2021). Contemporary narratives also mention a high intake of wild fruits, especially *mocán* (de Espinosa 1980).

### La Gomera

Seed remain analyses from La Gomera were carried out at five sites by means of systematic sampling and flotation. Only two, Lomito del Medio and El Alto del Garajonay, have yielded ^14^ C datings from a pre-Hispanic timeframe.

The evidence from Lomito del Medio consists of a group of charred *Hordeum* grains and a single grain of *Triticum* (Hernández-Marrero et al. 2016). Two ^14^ C dates respectively on *Triticum* and *Hordeum* grains place agricultural practices since the 5th–6th c. ce.

The assemblage from El Alto del Garajonay consists of only four *Hordeum* grains. The dating of two yielded a range between the 8th-11th c. ce. Most of the other seeds at this site are from wild plants such as *Ph. canariensis* (Morales et al. 2011).

Only *Hordeum* was identified at the remaining three sites (Cañada de la Gurona, Cueva Honda, and Sobrado de los Gomeros) (Hernández-Marrero et al. 2016) and no direct ^14^ C dates are currently available. The early European explorers cited *Hordeum* as the only crop cultivated by the indigenous occupants of La Gomera (known as *Gomeros*) (Frutuoso 1964).

### La Palma

Evidence of crops on La Palma is recorded at two rock shelters: El Tendal and Belmaco (Morales et al. 2013). The assemblage of El Tendal comprises *Hordeum*, *T. durum*, *L. culinaris* and *V. faba*. It is noteworthy that the dating of a *Hordeum* grain from the lower layers yielded 258–537 cal ce (Morales et al. 2013), the earliest range for a crop in the Canarian archipelago.

*Hordeum* is the only cultivated plant identified at Belmaco. It was collected in the lower levels of the stratigraphy and yielded a date ranging from the 7th–9th c. ce. The upper levels of the stratigraphy are characterised by the absence of crops and a presence especially of *O. europaea* ssp. *guanchica* and *Juniperus canariensis* (juniper) (Morales et al. 2013).

There is no indication among the European written records that the early population of La Palma cultivated crops. On the contrary, the first narratives suggest that the inhabitants (*Auharitas*) did not practice agriculture and only consumed the seeds of wild gathered plants such as *Cistus* sp. (de Abreu Galindo 1977).

### El Hierro

Crop cultivation evidence in El Hierro stems from three sites: La Lajura, Afotasa, and Hoya del Zarzal (Morales et al. 2017). The assemblage at La Lajura consists of a single *Hordeum* grain dated to the 5th–7th c. ce. The site is characterised by a larger presence of seeds of wild plants such as pine, *mocán* and *Retama rhodorhizoides* (white-broom) (Morales et al. 2017). The crop plants recovered at Afotasa include *Hordeum* and *Triticum* grains. The dating of a *Triticum* grain yielded a range covering the 6th–7th c. ce, while that of *Hordeum* spanned the 13th–14th c. ce (Morales et al. 2017). A single grain of *Hordeum* from Hoya del Zarzal, dates to between the 13th–14th c. ce (Morales et al. 2017).

Recent excavations at Cueva de la Herradura, a cave along the southern coast dated to the 3rd–7th c. ce, applied large-scale systematic sampling and sediment flotation techniques. However, the tests yielded no crops, only wild plant seeds such as *mocán, haya* (*Myrica faya*) and pine.

Written sources from the 15th c. allude to the cultivation of certain crops by the indigenous population (known as *Bimbapes*). Gaspar Frutuoso, a Portuguese explorer, cites *Hordeum* cultivation (Frutuoso 1964). French conquerors, in turn, allude to *Triticum* and *V. faba* but make no mention of *Hordeum* (Bontier and Le Verrier 1980).

## Discussion

### Origin and spread of agriculture

The Amazigh initially set foot in the archipelago between the 2nd and 5th c. ce and established permanent settlements on each of the different islands. The findings of this study suggest that the settlers introduced the following cultivated plants: *Hordeum*, *T. durum*, *L. culinaris*, *V. faba*, *P. sativum* and *F. carica*. The earliest reliable site yielding this crop ‘package’ (except *P. sativum* and *F. carica*) is El Tendal on La Palma dated to 258–537 cal ce (Morales et al. 2013). The second oldest set of dates (5th–8th c. ce) are from the eastern islands, notably from the site of Cueva de Villaverde on Fuerteventura. Here the set consists of *Triticum*, *Hordeum* and *Lens*. There is no archaeobotanical evidence of agriculture on Lanzarote during the 1st millennium ce due to a lack of systematic sampling and analyses of seed remains.

Genetic evidence obtained from analyses of currently cultivated *Hordeum* in Lanzarote indicates that local landraces diverged genetically from those of the western islands in at least 1,000 years BP, and from *Hordeum* from the African mainland more than 2,000 years ago. This suggests that the same *Hordeum* has been cultivated on Lanzarote since its initial colonisation (Hagenblad and Morales 2020). In addition, analyses of aDNA obtained from archaeological *Hordeum* grains from Gran Canaria reveal that this crop was probably introduced from north-western Africa, possibly current Morocco (Hagenblad et al. 2017). Linguistic analyses of plant names assigned to the Amazigh population of the Canary Islands suggest strong similarities with the current names used in the Amazigh languages of North Africa, further confirming a North African origin for the crop ‘package’ (Blench 2021).

The western islands of La Gomera and El Hierro also provide early archaeobotanical evidence of agriculture, notably *Hordeum* and *Triticum* from the 5th c. ce. The data available from the central islands of Gran Canaria and Tenerife suggest slightly younger dates. The earliest crops on Gran Canaria date from the 6th–7th c. ce, while those on Tenerife from the 7th–9th c. ce (Table 4). Moreover, cereal pollen is recorded on Gran Canaria in natural layers in a basin of volcanic origin dating to the 1st–2nd c. ce (de Nascimento et al. 2016). These factors, along with evidence of changes in vegetation and the clearing of forests gleaned from pollen research, are interpreted as evidence of an earlier colonisation of the island and a concomitant introduction of agriculture.

However, it should be noted that the radiocarbon datings stem from analyses carried out on bulk sediment samples and are less accurate than those obtained from seeds (Pardo-Gordó et al. 2022). Additionally, porous volcanic soils can suffer from pollen infiltration. Certain studies on the colonisation of other islands point to similar contrasts between the earliest dated cultural layers and earlier palaeoecological records (Leppard et al. 2022) indicating that the results, pending further data, must be interpreted with caution.

The ^14^ C dates bear witness to a colonisation of the archipelago over ca. 200–300 years, with a settling of the eastern islands taking place two or three centuries prior to the others (Atoche Peña and Ramírez Rodríguez 2017; Velasco-Vázquez et al. 2020). It is intriguing nonetheless that the current assemblage of ^14^ C analyses has yielded older dates for crops in the westernmost islands than in the more central islands of Gran Canaria and Tenerife. If crops along with people and domestic animals were to have spread from the African mainland in a step-by-step fashion from the eastern to the western islands, one would expect later dates from the western islands as they are farther from the point of origin. These results are interpreted as resulting from the drawbacks of the methodological differences of the studies of each island. The early dates from the western islands could in fact relate to taphonomic issues and not serve as evidence of an earlier colonisation. The ^14^ C datings from Gran Canaria and Tenerife on wood charcoals and human remains from sites not systematically sampled for plant remains point to a human presence on each of the islands since at least the 4th-5th c. ce (Velasco-Vázquez et al. 2020). Therefore, it is likely that the lack of earlier dates of crops from Lanzarote, Tenerife, and Gran Canaria finds its origin in preservation processes and the absence of systematic analyses and research programs, especially for sites from the 1st millennium ce.

### Crop diversity

The evidence currently available suggests significant differences among the crop ‘packages’ of the different islands. The results are likely affected by differences of conditions of preservation and the number of sites analysed. Gran Canaria is the best sampled island and the one revealing the entire crop ‘package’. In addition, cave-granaries on this island have yielded high numbers of seeds, possibly because these plant remains were preserved by desiccation. The sole means of preservation on the other islands is charring, which led to the preservation of low numbers. Lanzarote, at the other end of the scale with only a single systematically sampled site, has yielded only *Hordeum*. However, it is possible that the differences in the range of crops cultivated on each island was also linked to changes in the original crop ‘package’ and each island’s size and specific agricultural evolution.

*H. vulgare* ssp. *vulgare* (six-row hulled barley) is the most abundant and widespread crop of the archipelago. It is recorded on all islands and is the only cultivated plant identified systematically in all the sites of the study area. This suggests that it was the most important grain in the indigenous diet throughout the pre-Hispanic period, a notion bolstered by the narratives of most European explorers (de Abreu Galindo 1977; de Espinosa 1980). Moreover, *Hordeum* is more drought tolerant and, compared to *Triticum*, can be grown in poorer soils rendering it better adapted to the semi-arid conditions of the Canary Islands (Hagenblad et al. 2019).

*Triticum* is less frequent and has so far not been recorded on Lanzarote. Preservation of rachis segments at certain sites on La Palma (Morales et al. 2013) and Gran Canaria (Morales et al. 2014) allows its identification as *T. durum*. There is no evidence of the cultivation of *T. aestivum* and/or other wheat species during the pre-Hispanic period. Molecular analyses of *Triticum* grains dated to the colonial period (15th–17th c. ce) reveal that *T. aestivum* (bread wheat) was introduced in this timeframe (Oliveira et al. 2012). European explorers also noted the scant role of *Triticum* within the indigenous diet, stating that its consumption was in the form of whole grains cooked in soups (de Abreu Galindo 1977).

Pulses are less common and only identified on the islands of Fuerteventura, Gran Canaria, Tenerife and La Palma. *Lens culinaris* and *V. faba* are the most common, and *Pisum* is only known at four sites from Gran Canaria and Tenerife. European explorers only allude to the cultivation of *V. faba* and *P. sativum* during the period of contact (14th–15th c. ce).

The only fruit crop identified is *F. carica*, and it is strictly limited to Gran Canaria. The fact that it is exclusive to this island may relate to the practice here of artificial irrigation and lack of contacts with other islands (Morales and Gil 2014b). The 7th–8th c. ce dating of individuals with *F. carica* seeds embedded in their dental caries clearly indicates the plant’s early importance. The high prevalence of caries among the indigenous population of Gran Canaria has been linked to a high consumption of sugar-rich foodstuffs such as *F. carica* and cereals (Morales and Delgado-Darias 2007). Preservation of whole *F. carica* fruits in cave-granaries prove that they were stored in dried form for later consumption and were probably a staple (Morales and Gil 2014b; Henríquez-Valido et al. 2020).

There is no evidence of the introduction of new crops into the Canary Islands prior to the arrival of the Europeans. This suggests that the crop ‘package’ arrived in a single timeframe during the initial colonisation. This also supports the notion of an isolation of the archipelago until the arrival of the European seafarers.

**Fig. 2:**
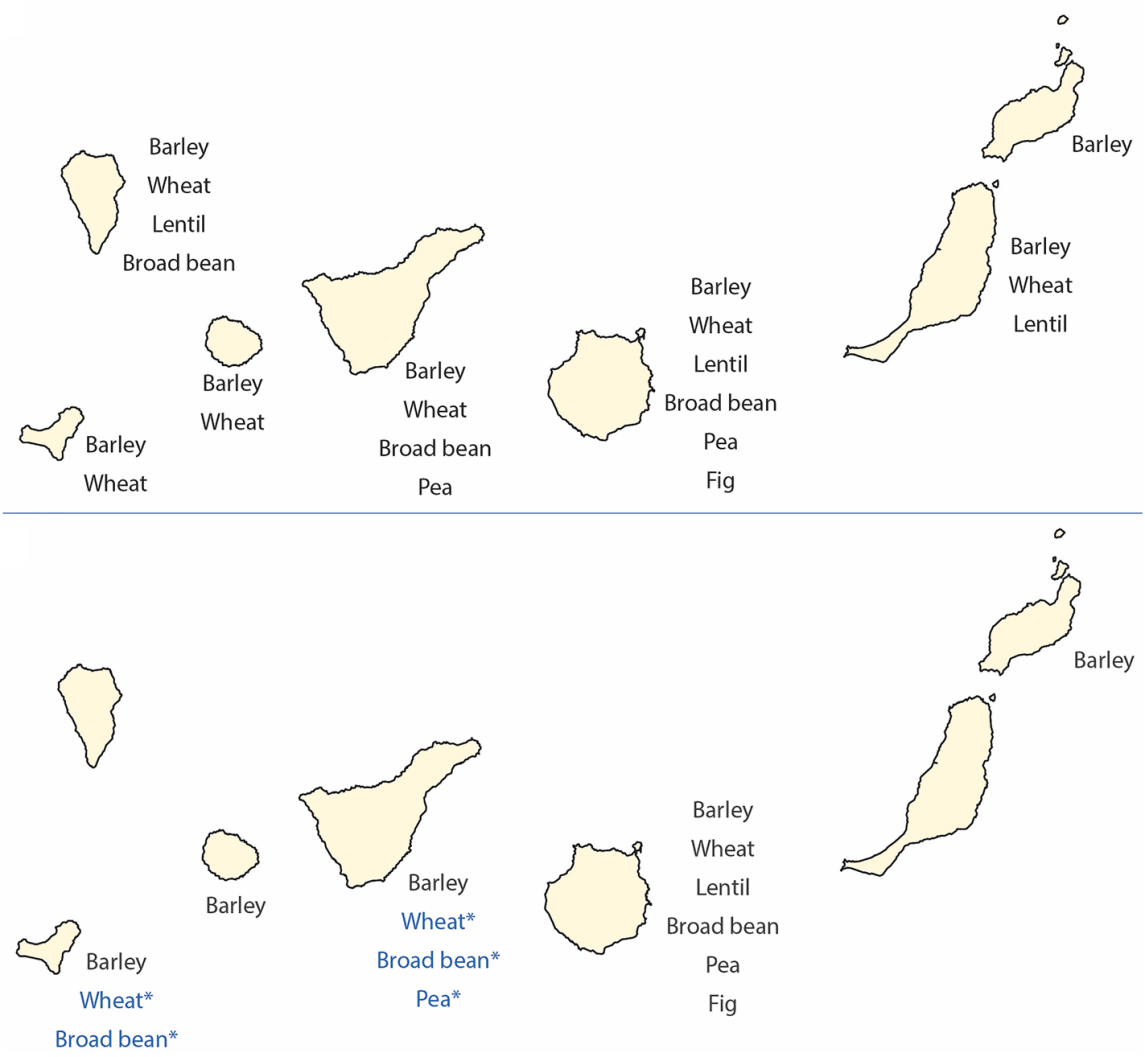
Maps of the Canary Islands indicating the crop plants present on each island: above during the 1st millennium ce and below during the 15th c. ce. The maps include data gleaned from archaeological evidence and old written narratives dating to the period of contact with the Europeans colonisers. *Crops only identified through the old written sources

The evidence currently available therefore suggests a decline in crop diversity over time on all the islands except Gran Canaria (Fig. 2). The most notable cases are La Palma and Fuerteventura where the crop ‘package’ was entirely lost by the 15th c. ce (Morales et al. 2013). *Triticum* in La Gomera and El Hierro is only recorded in contexts from the 1st millennium ce, and is absent from sites dated to the early 2nd millennium ce. The Cave of Don Gaspar of Tenerife reveals a similar pattern with *Triticum* and *V. faba* only associated with the older layers (Del Arco Aguilar et al. 1990). Although the results are still preliminary and possibly distorted, the loss of crop biodiversity is interpreted as stemming from the isolation of the different islands and the lack of means to restock lost or failed crops from other islands or the mainland. In addition, environmental pressures such as climatic fluctuations or volcanic eruptions probably favoured pastoralism, wild plant gathering and the exploitation of marine resources, reducing agriculture to a secondary role. Indeed, wild plant remains likely consumed by the indigenous populations are recorded on all islands except Lanzarote. Wild plants in fact are especially frequent at sites of the western islands where they most often outnumber their crop counterparts (Morales et al. 2017).

Gran Canaria, on the other hand, retained the entire crop ‘package’ throughout the pre-Hispanic period. The number and frequency of cultivated plants on this island is low in 1st millennium ce contexts. However, higher numbers of crop remains, especially *Hordeum* and *F. carica* seeds, appear in settlements from the 12th to the 15th c. ce (Morales 2010), concurring with an increase in capacity of storage features (Morales et al. 2018). These changes have been linked to agricultural and demographic growth (Morales 2010). Certain specialists suggest that the arrival of new migrants from North Africa could also have contributed to these social and economic shifts (Velasco-Vázquez et al. 2021; Alberto-Barroso et al. 2022a, 2022b). Different lines of evidence point to agriculture as one of the primary sources of food (Delgado-Darias et al. 2005, 2006; Arnay-de-la-Rosa et al. 2010), a hypothesis corroborated by historical written narratives penned by the first European explorers and colonists (de Abreu Galindo 1977).

Why was agriculture essential to Gran Canaria? It was continuously practiced on all the islands since the European conquest, potentially with a larger output on Tenerife than Gran Canaria during colonial and historical times (Hagenblad and Morales 2020). Tenerife is the largest island, and like most of the other western islands, it benefits from larger amounts of rain suggesting that precipitation does not directly explain this issue. Conversely, the orography of Gran Canaria, with its large valleys and permanent flows of water, is more suited to irrigated agriculture. Thus, the exploitation of irrigated fields in the large valleys yielded a more regular and reliable agricultural production than that of the other islands. No reference to pre-Hispanic irrigation has been recorded except on Gran Canaria. It is thus likely that the crops on the remaining islands were rainfed rendering them prone to harvest failures due to lack of sufficient precipitation. This likewise increased the risk of losing of crop biodiversity. Gran Canaria currently reveals no direct archaeological evidence of a pre-Hispanic irrigation system. However, recent analyses of historical archives confirm its existence during the period of contact with the Europeans (Díaz-Sierra 2022). In fact these archives signal that indigenous fields and irrigation canals were rapidly re-used by European settlers who replaced cereals, pulses and *F. carica* trees with *Saccharum* sp. (sugarcane) and other cash-crops introduced from Madeira and Europe (Díaz-Sierra 2022). On-going stable isotope analyses are expected to offer new evidence about agricultural practises such as artificial irrigation and manuring.

The abundance of cave-granaries intended for long-term storage of plant foods, only present in Gran Canaria, bolsters the significance of agricultural production on this island (Morales et al. 2014; Henríquez-Valido et al. 2020). Storage may have played a key role during years of crop failure due to extreme weather or plagues. In fact, European explorers mention the existence of the granaries in the 15th c. ce, stating that they served during famines to feed the population (Morales Padrón 2009).

## Conclusions

This review of the archaeobotanical data from the Canary Islands suggests the presence of a shared crop ‘package’ on the islands from at least the 3rd–5th c. ce. This set of plants consisting of *Hordeum vulgare* (hulled), *Triticum durum*, *Lens culinaris*, *Vicia faba*, *Pisum sativum* and *Ficus carica*, was, along with domesticated animals, most likely introduced from north-western Africa. It probably arrived on the islands at a single moment during the initial colonisation and was not modified or altered thereafter. The archipelago then remained isolated until the arrival of the European seafarers, this isolation leading to a decline in crop diversity over time on all islands except Gran Canaria. Agriculture on this island played a key role in the economy and society, and artificial irrigation of crops led to a more regular and predictable production. This, together with a superior capacity for stockpiling, led to larger and more sedentary populations than elsewhere in the archipelago, especially during the early 2nd millennium ce.

The different findings advanced here must be considered temporary as the data suffer from certain limitations and distortions due to the great disparities between the volume and number of samples of each site. Moreover, certain islands have benefitted from better study methods and offer more information than others. The current notions gleaned from this study should thus be considered as the groundwork for future research intended to fill the methodological gaps. To obtain more precise data, future excavations in the archipelago must, when appropriate, apply systematic soil sampling and water flotation processing techniques, and research programs need to focus on the smaller islands to compensate for their current lack of data.

**Table 4:** List of new radiocarbon dates on crop plants from archaeological sites in the Canary Islands

Island	Site	Taxon	Lab. code	^14^C yrs BP	Calibrated ages (ce)	
					68.2%	95.4%
El Hierro	Afotasa	*Hordeum*, gr	Beta-611193	690±30	1280–1382	1276–1390
Gran Canaria	Lomo San Pedro	*Homo sapiens*, bone	Beta-361285	790±30	1227–1269	1215–1280
Lanzarote	Fiquinineo	*Hordeum*, gr	Beta-561177	850±30	1166–1226	1054–1267
La Palma	El Tendal	*Hordeum*, gr	Beta-611189	950±30	1158–1219	1045–1228
Gran Canaria	Temisas	*H. sapiens*, bone	Beta-361283	1,150±30	883–976	774–992
Gran Canaria	Playa Chica	*Hordeum*, gr	Beta-593529	1,240±30	690–867	679–880
Gran Canaria	Acusa	*H. sapiens*, bone	Beta-361284	1,280±30	677–771	662–821
Fuerteventura	Cueva de Villaverde	*Hordeum*, gr	Beta-554548	1,300±30	669–772	660–774
Gran Canaria	El Draguillo	*H. sapiens*, bone	Beta-361286	1,430±30	605–647	584–658
La Palma	El Tendal	*Hordeum*, gr	Beta-611190	1,460±30	592–641	564–650
El Hierro	Afotasa	*Triticum*, gr	Beta-611194	1,480±30	568–636	550–644
La Gomera	Lomito de Enmedio	*Triticum*, gr	Beta-600220	1,530±30	482–595	434–603
La Gomera	Lomito de Enmedio	*Hordeum*, gr	Beta-600219	1,560±30	436–561	426–575
Fuerteventura	Cueva de Villaverde	*Triticum*, gr	Beta-554549	1,590±30	433–536	419–548

The list includes four on humans with *F. carica* seeds trapped in the dental caries of their teeth. The ranges are calibrated by means of the IntCal20 atmospheric calibration curve (Reimer et al. 2020) and the OxCal online software version 4.4. The two-sigma probability interval (95.4%) was applied when discussing the 14C ranges and the one-sigma probability interval (68.2%) was added to the dataset; for complete list see ESM

## Supplementary Information

Below is the link to the electronic supplementary material.

Supplementary material 1 (DOCX 35.5 kb)
