# Supplementary material for: Agriculture and crop dispersal in the western periphery of the Old World: the Amazigh/Berber settling of the Canary Islands (ca. 2nd–15th centuries ce)
Source: Veg Hist Archaeobot. 2023 Jun 22;35(1):219–33. doi: 10.1007/s00334-023-00920-6 (PMC12881054; doi:10.1007/s00334-023-00920-6)
Supplement: Supplementary file 1 — Supplementary material 1 (DOCX 35.5 kb) [file 334_2023_920_MOESM1_ESM.docx]

**ESM Table 1** List of radiocarbon dates on crop plants from archaeological sites in the Canary Islands. The list includes four on humans with fig seeds trapped in the dental caries of their teeth. The ranges are calibrated by means of the IntCal20 atmospheric calibration curve (Reimer et al. 2020) and the OxCal online software version 4.4. The two-sigma probability interval (95.4%) was applied when discussing the 14C ranges and the one-sigma probability interval (68.2%) was added to the dataset

| Island | Site | Taxon | Lab. code (Beta-) | ^14^C yrs BP | Calibrated ages (CE) | | Reference |
| --- | --- | --- | --- | --- | --- | --- | --- |
|  |  |  |  |  | 68.2% | 95.4% |  |
| Gran Canaria | Guayadeque | *Triticum*, gr | 269070 | 300±40 | 1515-1648 | 1480-1660 | Oliveira et al. 2012 |
| Tenerife | Chasogo | *Hordeum*, gr | 523054 | 340±30 | 1494-1631 | 1474-1638 | Morales et al. 2021 |
| Gran Canaria | Guayadeque | *Triticum*, gr | 269069 | 390±40 | 1448-1620 | 1437-1635 | Oliveira et al. 2012 |
| Gran Canaria | Cueva Pintada | *Hordeum*, gr | 209281 | 430±40 | 1428-1487 | 1415-1623 | Morales 2010 |
| Gran Canaria | Lomo Los Gatos | *Hordeum*, gr | 209728 | 430±40 | 1428-1487 | 1415-1623 | Morales 2010 |
| Gran Canaria | Cueva Pintada | *Hordeum*, gr | 209282 | 440±40 | 1426-1476 | 1408-1620 | Morales 2010 |
| Gran Canaria | Lomo Los Gatos | *Hordeum*, gr | 209729 | 450±40 | 1423-1466 | 1406-1618 | Morales 2010 |
| Tenerife | Chinguaro | *Hordeum*, gr | 261242 | 450±40 | 1423-1466 | 1406-1618 | Morales et al. 2017 |
| Gran Canaria | Temisas | *Hordeum*, gr | 362109 | 510±30 | 1409-1435 | 1328-1449 | Henríquez-Valido et al. 2019 |
| Gran Canaria | Cenobio de Valeron | *Hordeum*, gr | 384697 | 520±30 | 1405-1432 | 1327-1444 | Morales et al. 2018 |
| Gran Canaria | Temisas | *Hordeum*, gr | 362113 | 520±30 | 1405-1432 | 1327-1444 | Henríquez-Valido et al. 2019 |
| Gran Canaria | Acusa | *Hordeum*, gr | 317651 | 540±30 | 1329-1428 | 1322-1437 | Morales et al. 2014 |
| Gran Canaria | Temisas | *Hordeum*, gr | 362112 | 550±30 | 1328-1423 | 1318-1434 | Henríquez-Valido et al. 2019 |
| Gran Canaria | Cuevas Muchas | *Hordeum*, gr | 362104 | 560±30 | 1326-1415 | 1312-1428 | Hagenblad et al. 2017 |
| Gran Canaria | Acusa | *T. durum*, rachis | 317650 | 600±30 | 1326-1415 | 1312-1428 | Morales et al. 2014 |
| Gran Canaria | Cenobio de Valeron | *Hordeum*, gr | 390473 | 600±30 | 1328-1423 | 1318-1434 | Naranjo and Rodríguez 2015 |
| Gran Canaria | Temisas | *Hordeum*, gr | 362110 | 610±30 | 1314-1398 | 1301-1408 | Henríquez-Valido et al. 2019 |
| Gran Canaria | Cueva Pintada | *Hordeum*, gr | 209280 | 610±40 | 1306-1396 | 1299-1404 | Morales 2010 |
| Gran Canaria | Cuevas Muchas | *Hordeum*, gr | 362106 | 630±30 | 1305-1397 | 1293-1409 | Hagenblad et al. 2017 |
| El Hierro | Hoya del Zarzal | *Hordeum*, gr | 173702 | 650±40 | 1300-1393 | 1293-1398 | Morales et al. 2017 |
| Gran Canaria | La Fortaleza | *Hordeum*, gr | 477345 | 660±30 | 1291-1390 | 1280-1397 | Henríquez-Valido et al. 2020 |
| Gran Canaria | Cenobio de Valeron | *Hordeum*, gr | 384696 | 670±30 | 1284-1384 | 1277-1392 | Morales et al. 2018 |
| Gran Canaria | La Fortaleza | *Hordeum*, gr | 477344 | 670±30 | 1284-1384 | 1277-1392 | Henríquez-Valido et al. 2020 |
| Gran Canaria | La Fortaleza | *Hordeum*, gr | 477346 | 670±30 | 1287-1387 | 1279-1394 | Henríquez-Valido et al. 2020 |
| Gran Canaria | La Fortaleza | *Hordeum*, gr | 477348 | 680±30 | 1284-1384 | 1277-1392 | Henríquez-Valido et al. 2020 |
| Gran Canaria | La Fortaleza | *V. faba*, seed | 512953 | 690±30 | 1279-1377 | 1272-1389 | Moreno-Benitez et al. 2022 |
| El Hierro | Afotasa | *Hordeum*, gr | 611193 | 690±30 | 1280-1382 | 1276-1390 | This study |
| Gran Canaria | Cuevas Muchas | *Hordeum*, gr | 362105 | 710±30 | 1279-1377 | 1272-1389 | Hagenblad et al. 2017 |
| Gran Canaria | La Fortaleza | *Hordeum*, gr | 477350 | 760±30 | 1273-1299 | 1262-1387 | Henríquez-Valido et al. 2020 |
| Gran Canaria | Cenobio de Valeron | *Hordeum*, gr | 390474 | 780±30 | 1229-1274 | 1219-1280 | Morales et al. 2018 |
| Tenerife | Cruz de Tea | *Hordeum*, gr | 477366 | 780±30 | 1231-1281 | 1222-1285 | Morales et al. 2021 |
| Gran Canaria | La Fortaleza | *Hordeum*, gr | 347796 | 790±30 | 1227-1269 | 1215-1280 | Morales et al. 2018 |
| Gran Canaria | Lomo San Pedro | *Homo sap.*, bone | 361285 | 790±30 | 1227-1269 | 1215-1280 | This study |
| Gran Canaria | La Fortaleza | *Hordeum*, gr | 554542 | 800±30 | 1225-1264 | 1180-1279 | Henríquez-Valido et al. 2020 |
| Gran Canaria | La Fortaleza | *Hordeum*, gr | 477351 | 810±30 | 1221-1264 | 1178-1276 | Henríquez-Valido et al. 2020 |
| Gran Canaria | Cendro | *Hordeum*, gr | 330597 | 840±30 | 1176-1259 | 1162-1267 | Morales et al. 2017 |
| Lanzarote | Fiquinineo | *Hordeum*, gr | 561177 | 850±30 | 1166-1226 | 1054-1267 | This study |
| Gran Canaria | Acusa | *Hordeum*, gr | 362103 | 860±30 | 1167-1221 | 1052-1263 | Hagenblad et al. 2017 |
| Gran Canaria | Temisas | *Hordeum*, gr | 362111 | 860±30 | 1167-1221 | 1052-1263 | Henríquez-Valido et al. 2019 |
| Gran Canaria | Cenobio de Valeron | *F. carica*, seed | 384698 | 890±30 | 1163-1219 | 1047-1261 | Morales et al. 2018 |
| Gran Canaria | La Fortaleza | *L. culinaris*, seed | 477347 | 950±30 | 1054-1215 | 1045-1223 | Henríquez-Valido et al. 2020 |
| La Palma | El Tendal | *Hordeum*, gr | 611189 | 950±30 | 1158-1219 | 1045-1228 | This study |
| Gran Canaria | Temisas | *Hordeum*, gr | 469050 | 960±30 | 1038-1152 | 1028-1162 | Henríquez-Valido et al. 2019 |
| Gran Canaria | La Cerera | *Hordeum*, gr | 317655 | 980±30 | 1034-1151 | 1025-1160 | Morales et al. 2017 |
| Gran Canaria | Cendro | *Hordeum*, gr | 330595 | 980±30 | 1038-1152 | 1028-1162 | Morales et al. 2017 |
| Gran Canaria | Temisas | *Hordeum*, gr | 469049 | 1,100±30 | 987-1026 | 895-1035 | Henríquez-Valido et al. 2019 |
| Gran Canaria | Dunas de Maspalomas | *V. faba*, seed | 359511 | 1,120±30 | 896-992 | 887-1017 | Rodríguez-Rodríguez et al. 2021 |
| La Gomera | Alto del Garajonay | *Hordeum*, gr | 206015 | 1,130±40 | 893-977 | 774-995 | Morales et al. 2011 |
| Gran Canaria | La Fortaleza | *Hordeum*, gr | 477349 | 1,140±30 | 885-986 | 773-1014 | Henríquez-Valido et al. 2020 |
| Gran Canaria | Temisas | *H. sapiens*, bone | 361283 | 1,150±30 | 883-976 | 774-992 | This study |
| Tenerife | Bencomo | *Hordeum*, gr | 523055 | 1,180±30 | 775-891 | 771-973 | Marrero Salas et al. 2021 |
| Gran Canaria | Dunas de Maspalomas | *Hordeum*, gr | 359513 | 1,200±30 | 782-881 | 706-945 | Rodríguez-Rodríguez et al. 2021 |
| Gran Canaria | La Montañeta | *Hordeum*, gr | 298966 | 1,220±30 | 784-878 | 687-888 | Morales et al. 2018 |
| Gran Canaria | Playa Chica | *Hordeum*, gr | 593529 | 1,240±30 | 690-867 | 679-880 | This study |
| La Palma | Belmaco | *Hordeum*, gr | 206151 | 1,250±40 | 681-826 | 671-880 | Morales et al. 2013 |
| Tenerife | Chinguaro | *Hordeum*, gr | 261243 | 1,260±40 | 677-820 | 666-877 | Morales et al. 2017 |
| Gran Canaria | Acusa | *H. sapiens*, bone | 361284 | 1,280±30 | 677-771 | 662-821 | This study |
| Fuerteventura | Cueva de Villaverde | *Hordeum*, gr | 554548 | 1,300±30 | 669-772 | 660-774 | This study |
| La Palma | El Tendal | *Hordeum*, gr | 206155 | 1,400±40 | 606-661 | 575-758 | Morales et al. 2013 |
| Gran Canaria | La Cerera | *Triticum*, gr | 302329 | 1,410±30 | 607-655 | 597-664 | Morales et al. 2017 |
| Gran Canaria | El Draguillo | *H. sapiens*, bone | 361286 | 1,430±30 | 605-647 | 584-658 | This study |
| La Palma | El Tendal | *Hordeum*, gr | 611190 | 1,460±30 | 592-641 | 564-650 | This study |
| El Hierro | Afotasa | *Triticum*, gr | 611194 | 1,480±30 | 568-636 | 550-644 | This study |
| La Gomera | Lomito de Enmedio | *Triticum*, gr | 600220 | 1,530±30 | 482-595 | 434-603 | This study |
| El Hierro | La Lajura | *Hordeum*, gr | 261244 | 1,530±40 | 440-598 | 430-633 | Morales et al. 2017 |
| La Gomera | Lomito de Enmedio | *Hordeum*, gr | 600219 | 1,560±30 | 436-561 | 426-575 | This study |
| La Palma | El Tendal | *Hordeum*, gr | 206156 | 1,570±40 | 434-548 | 418-577 | Morales et al. 2013 |
| Fuerteventura | Cueva de Villaverde | *Triticum*, gr | 554549 | 1,590±30 | 433-536 | 419-548 | This study |
| La Palma | El Tendal | *Hordeum*, gr | 206154 | 1,660±40 | 263-527 | 258-537 | Morales et al. 2013 |
| Gran Canaria | La Fortaleza | *V. faba*, seed | 032135* | 1,078±24 | 900-1017 | 894-1023 | Moreno-Benítez et al. 2022 |
| Gran Canaria | Dunas de Maspalomas | *Hordeum*, gr | 359512 | 1,050±30 | 987-1026 | 895-1035 | Rodríguez-Rodríguez et al. 2021 |
| Gran Canaria | La Cerera | *Hordeum*, gr | 302328 | 1,050±30 | 987-1026 | 895-1035 | Morales et al. 2017 |
| Gran Canaria | Cendro | *Hordeum*, gr | 330596 | 1,050±30 | 902-1035 | 892-1150 | Morales et al. 2017 |
| La Gomera | Alto del Garajonay | *Hordeum*, gr | 206018 | 1,040±40 | 1024-1149 | 995-1158 | Morales et al. 2011 |

*D-AMS

References

Hagenblad J, Morales J, Leino MW, Rodríguez-Rodríguez AC (2017) Farmer fidelity in the Canary Islands revealed by ancient DNA from prehistoric seeds. J Archaeol Sci 78:78-87. https://doi.org/10.1016/j.jas.2016.12.001

Henríquez-Valido P, Morales J, Vidal-Matutano P et al (2019) Arqueoentomología y arqueobotánica de los espacios de almacenamiento a largo plazo: el granero de Risco Pintado, Temisas (Gran Canaria). Trab Prehist 76:120-137. <https://doi.org/10.3989/tp.2019.12229>

Henríquez-Valido P, Morales J, Vidal-Matutano P et al (2020) Archaeoentomological indicators of long-term food plant storage at the Prehispanic granary of La Fortaleza (Gran Canaria, Spain). J Archaeol Sci 120:105179. https://doi.org/10.1016/j.jas.2020.105179

Marrero-Salas E, Ruiz González H, García Ávila JC et al (2021) Las Cuevas de Bencomo (La Orotava, Tenerife). De la historiografía al dato arqueológico. Vegueta Anu Fac Geogr Hist 21:499-530. <https://doi.org/10.51349/veg.2021.1.19>

Morales J (2010) El uso de las plantas en la prehistoria de Gran Canaria: alimentación, agricultura y ecología. Cabildo de Gran Canaria, Las Palmas de Gran Canaria

Morales J, Henríquez-Valido P, Moreno-Benítez M et al (2018) Du laurier dans les greniers de Grande Canarie. Tech Cult 69:126–129. <https://doi.org/10.4000/tc.8930>

Morales J, Navarro-Mederos JF, Rodríguez-Rodríguez A (2011) Plant offerings to the gods: seed remains from a pre-Hispanic sacrificial altar in La Gomera Island (Canary Islands, Spain). In: Fahmy AG, Kahlheber S, D’Andrea AC (eds) Windows on the African Past: Current approaches to African archaeobotany. Africa Magna Verlag, Frankfurt am Main, pp 67-78

Morales J, Rodríguez-Rodríguez A, González-Marrero MC et al (2014) The archaeobotany of long-term crop storage in northwest African communal granaries: a case study from pre-Hispanic Gran Canaria (cal. AD 1000–1500). Veget Hist Archaeobot 23:789-804. https://doi.org/10.1007/s00334-014-0444-4

Morales J, Vidal-Matutano P, Marrero-Salas E et al (2021) High-mountain plant use and management: macro-botanical data from the pre-Hispanic sites of Chasogo and Cruz de Tea, 13–17th centuries AD, Tenerife (Canary Islands, Spain). J Archaeol Sci Rep 35:102730. <https://doi.org/10.1016/j.jasrep.2020.102730>

Morales J, Rodríguez A, Henríquez-Valido P (2017) Agricultura y recolección vegetal en la arqueología prehispánica de las Islas Canarias (siglos III-XV d.C.): la contribución de los estudios carpológicos. In: Fernández Eraso J, Mujika Alustiza JA, Arrizabalaga Valbuena A, García Diez M (eds) Miscelánea en homenaje a Lydia Zapata Peña (1965-2015). Universidad del País Vasco, Vitoria, pp 189-218

Morales J, Rodríguez-Rodríguez AC, Marrero Á (2013) Prehistoric Plant Use on La Palma Island (Canary Islands, Spain): an example of the disappearance of agriculture in an isolated environment. In: Stevens CJ, Nixon S, Murray MA, Fuller DQ (eds) Archaeology of African Plant Use. Left Coast Press, Walnut Creek, pp 195-204

Moreno-Benítez MA, Velasco-Vázquez J, Alberto-Barroso V, Delgado-Darias T (2022) ¿Poblamiento y cambio social de un territorio aislado? Propuestas sobre la evolución de la ocupación territorial de la isla de Gran Canaria en época prehispánica. Zephyrvs 89:213-235. <https://doi.org/10.14201/zephyrus202289213235>

Naranjo-Mayor Y, Rodríguez-Rodríguez A (2015) Artefactos e instrumentos de piedra en un espacio de almacenamiento colectivo. El caso de El Cenobio de Valerón (Gran Canaria, España). Munibe Antropol-Arkeol 66:291-308

Oliveira HR, Civáň P, Morales J et al (2012) Ancient DNA in archaeological wheat grains: Preservation conditions and the study of pre-Hispanic agriculture on the island of Gran Canaria (Spain). J Archaeol Sci 39:828-835. https://doi.org/10.1016/j.jas.2011.10.008

Rodríguez-Rodríguez A, Santana-Cabrera J, Castellano Alonso P et al (2021) Un lugar entre las dunas. Aprovechamiento oportunista de un espacio costero durante la etapa preeuropea de la isla de Gran Canaria (circa siglos VIII-XI AD). Trab Prehist 78:325-343. https://doi.org/10.3989/tp.2021.12279
